# Dicke model

**DOI:** 10.1371/journal.pone.0235197

**Published:** 2020-09-04

**Authors:** Mor M. Roses, Emanuele G. Dalla Torre

**Affiliations:** Department of Physics, Bar-Ilan University, Ramat Gan, Israel; Rice University, UNITED STATES

## Abstract

The **Dicke model** is a fundamental model of quantum optics, which describes the interaction between light and matter. In the Dicke model, the *light* component is described as a single quantum mode, while the *matter* is described as a set of two-level systems. When the coupling between the light and matter crosses a critical value, the Dicke model shows a mean-field phase transition to a superradiant phase. This transition belongs to the Ising universality class and was realized experimentally in cavity quantum electrodynamics experiments. Although the superradiant transition bears some analogy with the lasing instability, these two transitions belong to different universality classes.

## The model and its symmetries

The Dicke model is a quantum mechanical model that describes the coupling between a single-mode cavity and *N* two-level systems, or equivalently *N* spin-½ degrees of freedom. The model was first introduced in 1973 by K. Hepp and E. H. Lieb [1]. Their study was inspired by the pioneering work of R. H. Dicke on the superradiant emission of light in free space [2] and named after him.

Like any other model in quantum mechanics, the Dicke model includes a set of quantum states (the Hilbert space) and a total-energy operator (the Hamiltonian). The **Hilbert space** of the Dicke model is given by (the tensor product of) the states of the cavity and of the two-level systems. The Hilbert space of the cavity can be spanned by Fock states with *n* photons, denoted by |*n*⟩. These states can be constructed from the vacuum state |*n* = 0⟩ using the canonical ladder operators, *a*^†^ and *a*, which add and subtract a photon from the cavity, respectively. The states of each two-level system are referred to as *up* and *down* and are defined through the **spin** operators $\vec{\sigma_{j}}=(\sigma_{j}^{x},\sigma_{j}^{y},\sigma_{j}^{z})$, satisfying the spin algebra $[\sigma_{j}^{x},\sigma_{k}^{y}]=i\hbar \sigma_{j}^{z}\delta_{i,k}$. Here ℏ is the Planck constant and *j* = (0,1,2,…,*N*) indicates a specific two-level system. (Note that the spin operators are often represented Pauli matrices $\tilde{\sigma }$ through the relation $\sigma^{\alpha }=\hbar {\tilde{\sigma }}^{\alpha }$. In some References, the Hamiltonian of the Dicke model is represented in terms of Pauli matrices, rather than spin operators).

The **Hamiltonian** of the Dicke model is

$$H=\hbar \omega_{c}a^{†}a+\omega_{z}\sum_{j=0}^{N}\sigma_{j}^{z}+\frac{2\lambda }{\sqrt{N}}(a+a^{†})\sum_{j}\sigma_{j}^{x}.$$ (1)

Here, the first term describes the energy of the cavity and equals to the product of the energy of a single cavity photon ℏ*ω*_*c*_ (where *ω*_*c*_ is the cavity frequency), times the number of photons in the cavity, *n*_*c*_ = *a*^†^*a*. The second term describes the energy of the two-level systems, where ℏ*ω*_*z*_ is the energy difference between the states of each two-level system. The last term describes the coupling between the two-level systems and the cavity and is assumed to be proportional to a constant, *λ*, times the inverse of the square root of the number of two-level systems. This assumption allows one to obtain a phase transition in the limit of *N*→∞ (see below). The coupling can be written as the sum of two terms: a *co-rotating* term that conserves the number of excitations and is proportional to *aσ*^+^+*a*^†^*σ*^−^ and a *counter-rotating* term proportional to *aσ*^−^+*a*^†^*σ*^+^, where *σ*^±^ = *σ*^*x*^±*iσ*^*y*^ are the spin ladder operators.

The **Hamiltonian** in (Eq 1) assumes that that all the spins are identical (i.e. have the same energy difference and are equally coupled to the cavity). Under this assumption, one can define the macroscopic spin operators $S^{\alpha }=\sum_{j=0}^{N}\sigma_{j}^{\alpha }$, with *α* = *x*,*y*,*z*, which satisfy the spin algebra, [*S*^*x*^,*S*^*y*^] = *i*ℏ*S*^*z*^. Using these operators, one can rewrite the **Hamiltonian** (Eq 1) as

$$H=\hbar \omega_{c}+\omega_{z}S^{z}+\frac{2\lambda }{\sqrt{N}}(a+a^{†})S^{x}.$$ (2)

This notation simplifies the numerical study of the model because it involves a single spin-S with *S*≤*N*/2, whose Hilbert space has size 2*S*+1, rather than *N* spin-1/2, whose Hilbert space has size 2^*N*^.

The Dicke model has one **global symmetry**,

$$P:(a,\sigma^{\pm })\to (-a,-\sigma^{\pm }).$$ (3)

Because P squares to unity (i.e. if applied twice, it brings each state back to its original state), it has two eigenvalues, 1 and −1. This symmetry is associated with a conserved quantity: the parity of the total number of excitations, $P={\left(-1\right)}^{N_{ex}}$ where

$$N_{ex}=a^{†}a+\sum_{j=0}^{N}\sigma_{j}^{z}.$$ (4)

This parity conservation can be seen from the fact that each term in the Hamiltonian preserves the excitation number, except for the counter-rotating terms, which can only change the excitation number by ±2. A state of the Dicke model is said to be *normal* when this symmetry is preserved, and *superradiant* when this symmetry is spontaneously broken.

### Related models

The Dicke model is closely related to other models of quantum optics. Specifically, the Dicke model with a single two-level system, *N* = 1, is called the Rabi model. In the absence of counter-rotating terms, the model is called Jaynes-Cummings for *N* = 1 and Tavis-Cummings for *N*>1. These two models conserve the number of excitations *N*_ex_ and are characterized by a *U*(1) symmetry. The spontaneous breaking of this symmetry gives rise to a lasing state (see below).

The relation between the Dicke model and other models is summarized in Table 1 [3].

**Table 1:** Relation between the Dicke model and other models.

Model’s name	Counter-rotating terms?	Symmetry	Number of two-level systems
Jaynes-Cummings	No	*U*(1)	*N* = 1
Tavis-Cummings	No	*U*(1)	*N*>1
Rabi model	Yes	P	*N* = 1
Dicke	Yes	P	*N*>1

## The superradiant phase transition

Early studies of the Dicke model considered its equilibrium properties [1]. These works considered the limit of *N*→∞ (also known as the *thermodynamic limit*) and assumed a thermal partition function, *Z* = exp(−*H*/*k*_*B*_*T*), where *k*_*B*_ is the Boltzmann constant and *T* is the temperature. It was found that, when the coupling *λ* crosses a critical value *λ*_*c*_, the Dicke model undergoes a second-order phase transition, known as the superradiant phase transition. In their original derivation, Hepp and Lieb [1] neglected the effects of counter-rotating terms and, thus, actually considered the Tavis-Cummings model (see above). Further studies of the full Dicke model found that the phase transition still occurs in the presence of counter-rotating terms, albeit at a different critical coupling [4].

**Fig 1:**
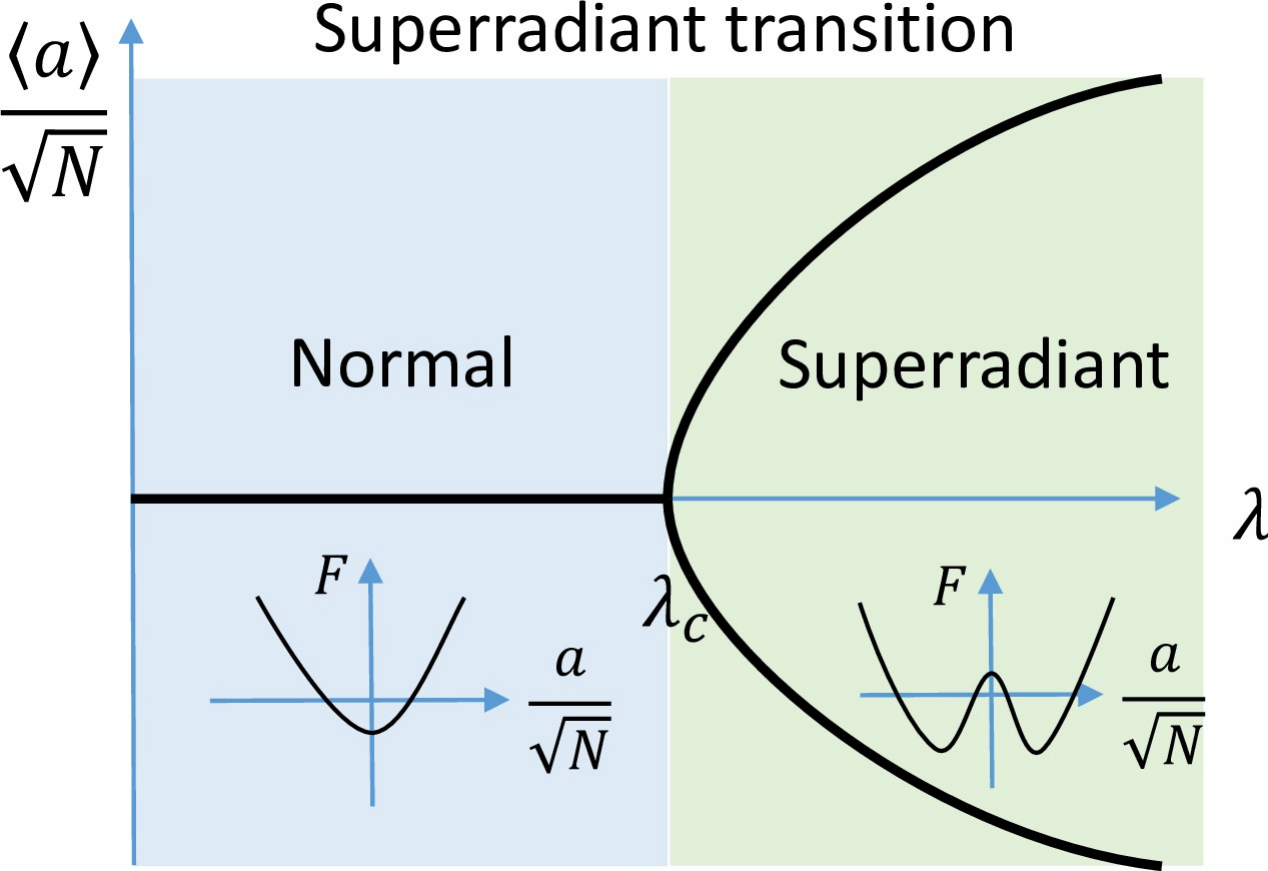
Schematic plot of the order parameter of the Dicke transition, which is zero in the normal phase and finite in the superradiant phase. The inset shows the free energy in the normal and superradiant phases, see Eq 5.

The superradiant transition spontaneously breaks the parity symmetry, P, defined in Eq 3. The order parameter of this phase transition is $\langle a\rangle /\sqrt{N}$. In the thermodynamic limit, this quantity tends to zero if the system is normal, or to one of two possible values, if the system is superradiant. These two values correspond to physical states of the cavity field with opposite phases (see Eq 3 and, correspondingly, to states of the spin with opposite *x* components). Close to the superradiant phase transition, the order parameter depends on *λ* as $\langle a\rangle /\sqrt{N}∼{\left(\lambda_{c}-\lambda \right)}^{-1/2}$ (see **Fig 1**). This dependence corresponds to the mean-field critical exponent *β* = 1/2.

## Mean-field description of the transition

The simplest way to describe the superradiant transition is to use a mean-field approximation, in which the cavity field operators are substituted by their expectation values. Under this approximation, which is exact in the thermodynamic limit, the Dicke Hamiltonian of Eq 1 becomes a sum of independent terms, each acting on a different two-level system, which can be diagonalized independently. At thermal equilibrium (see above), one finds that the free energy per two-level system is [5]

$$F\left(\frac{\left\langle a\right\rangle }{\sqrt{N}}=\alpha \right)=\omega_{c}\alpha^{2}-k_{B}T\;\ln\left(2\;\cosh\left(\frac{\sqrt{\omega_{z}^{2}+16\lambda^{2}\alpha^{2}}}{2k_{B}T}\right)\right).$$ (5)

The critical coupling of the transition can be found by the condition *dF*/*dα*(*α* = 0) = 0, leading to

$$\lambda_{c}=\frac{1}{2}\sqrt{\omega_{c}\omega_{z}\;\coth\left(\frac{\hbar \omega_{z}}{2k_{B}T}\right)}.$$ (6)

For *λ*<*λ*_*c*_ *F* has one minimum, while for *λ*>*λ*_*c*_, it has two minima (see the inset of **Fig 1**). In the limit of *T*→0 one obtains an expression for the critical coupling of the zero-temperature superradiant phase transition, $\lambda_{c}=\sqrt{\omega_{c}\omega_{z}}/2$.

## The open Dicke model

The Dicke model of Eq 1 assumes that the cavity mode and the two-level systems are perfectly isolated from the external environment. In actual experiments, this assumption is not valid: the coupling to free modes of light can cause the loss of cavity photons and the decay of the two-level systems (i.e. dissipation channels). It is worth mentioning, that these experiments use driving fields (e.g. laser fields) to implement the coupling between the cavity mode and the two-level systems. The various dissipation channels can be described by adding a coupling to additional environmental degrees of freedom. By averaging over the dynamics of these external degrees of freedom one obtains equations of motion describing an open quantum system. According to the common Born-Markov approximation, one can describe the dynamics of the system with the quantum master equation in Lindblad form [6]

$$\frac{d\rho }{dt}=-\frac{i}{\hbar }[H,\rho ]+\sum_{\alpha }\gamma_{\alpha }(L_{\alpha }\rho L_{\alpha }^{†}-\frac{1}{2}\left\{L_{\alpha }^{†}L_{\alpha },\rho \right\}).$$ (7)

Here, *ρ* is the density matrix of the system, *L*_*α*_ is the Lindblad operator of the decay channel *α*, and *γ*_*α*_ the associated decay rate. When the Hamiltonian *H* is given by Eq 1, the model is referred to as the open Dicke model.

Some common decay processes that are relevant to experiments are given in Table 2.

**Table 2:** Common decay processes.

-	Cavity decay	Atomic decay	Atomic dephasing	Collective decay
Lindbladian	*L* = *a*	$L=\sigma_{i}^{-}$	$L=\sigma_{i}^{z}$	$L=\sum_{i}\sigma_{i}^{-}$
Decay rate	*κ*	*γ*_↓_	*γ*_*ϕ*_	Γ_↓_

In the theoretical description of the model, one often considers the steady state where *dρ*/*dt* = 0. In the limit of *N*→∞, the steady state of the open Dicke model shows a continuous phase transition, often referred to as the *nonequilibrium superradiant transition*. The critical exponents of this transition are the same as the equilibrium superradiant transition at finite temperature (and differ from the superradiant transition at zero temperature).

## The superradiant transition and Dicke superradiance

**Fig 2:**
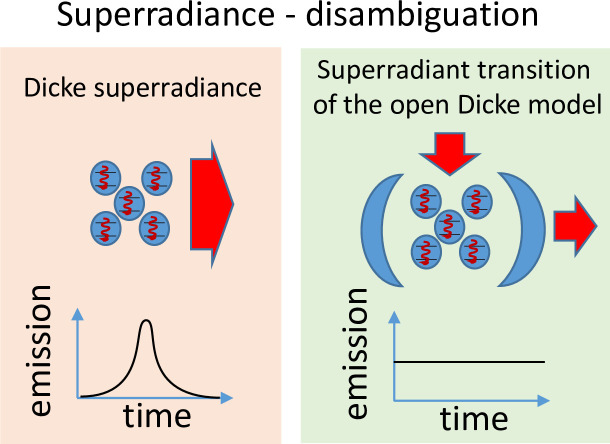
Schematic representation of the difference between Dicke superradiance and the superradiant transition of the open Dicke model.

The superradiant transition of the open Dicke model is related to, but differs from, Dicke superradiance (see **Fig 2**).

Dicke superradiance is a collective phenomenon in which many two-level systems emit photons coherently in free space [2, 7]. It occurs if the two-level systems are initially prepared in their excited state and placed at a distance much smaller than the relevant photon's wavelength. Under these conditions, the spontaneous decay of the two-level systems becomes much faster: the two-level systems emit a short pulse of light with large amplitude. Under ideal conditions, the pulse duration is inversely proportional to the number of two-level systems, *N*, and the maximal intensity of the emitted light scales as *N*^2^. This is in contrast to the spontaneous emission of *N* independent two-level systems, whose decay time does not depend on *N* and where the pulse intensity scales as *N*.

As explained above, the open Dicke model rather models two-level systems coupled to a quantized cavity and driven by an external pump (see **Fig 2**). In the normal phase, the intensity of the cavity field does not scale with the number of atoms *N*, while in the superradiant phase, the intensity of the cavity field is proportional to ⟨*a*^†^*a*⟩~*N*.

The scaling laws of Dicke superradiance and of the superradiant transition of the Dicke model are summarized in Table 3.

**Table 3:** Scaling laws of Dicke superradiance and of the superradiant transition of the Dicke model.

	Dicke superradiance [2]	Superradiant transition of the Dicke model [1]
Environment	Free space	Cavity
Duration	Transient	Steady state
Intensity of the field (normal)	*N*	1
Intensity of the field (superradiant)	*N*^2^	*N*

## Experimental realizations of the Dicke model

The simplest realization of the Dicke model involves the dipole coupling between two-level atoms in a cavity (see **Fig 2**, right panel). In this system, the observation of the superradiant transition is hindered by two possible problems: (1) The bare coupling between atoms and cavities is usually weak and insufficient to reach the critical value *λ*_*c*_, see Eq 6 [8]. (2) An accurate modelling of the physical system requires to consider *A*^2^ terms that according to a *no-go theorem*, may prevent the transition. Both limitations can be circumvented by applying external pumps on the atoms and creating an effective Dicke model in an appropriately rotating frame [9, 10].

**Fig 3:**
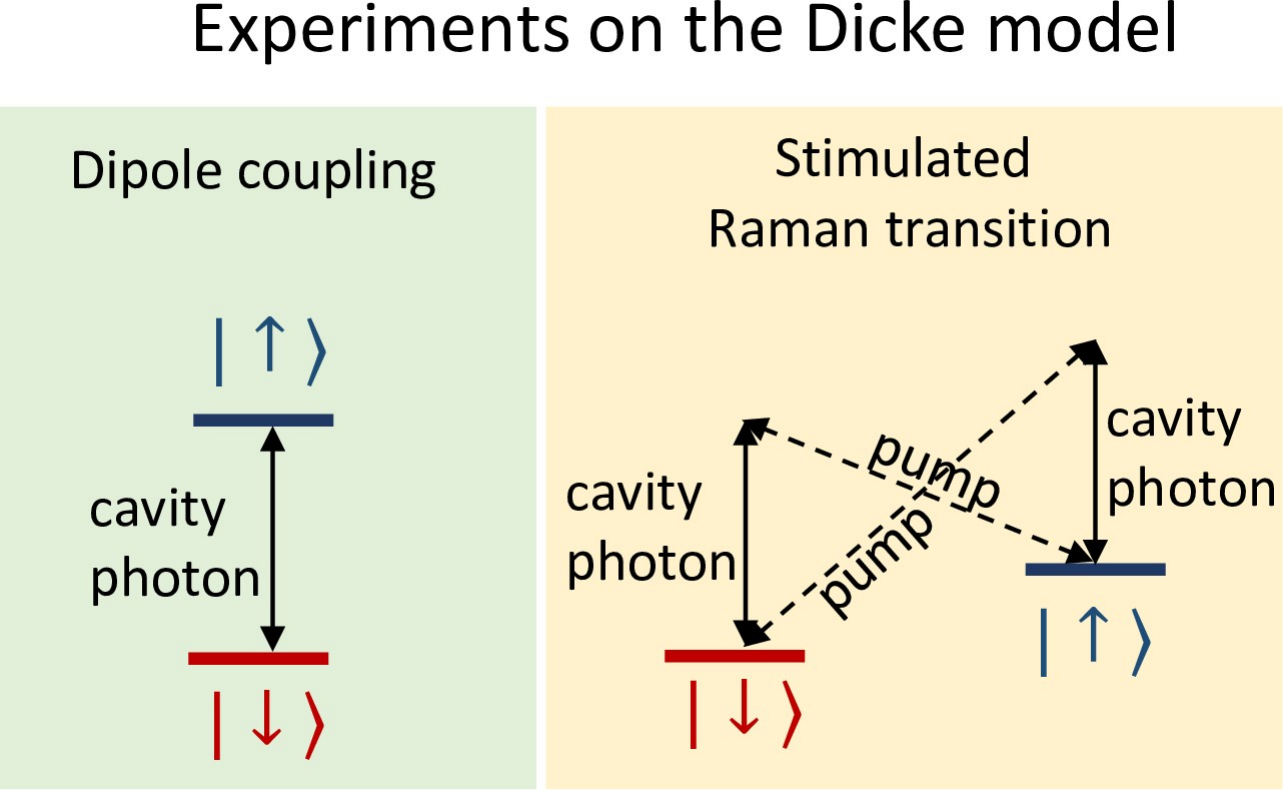
**Schematic representation of two schemes to experimentally realize the Dicke model: On the left, the equilibrium approach based on the dipole coupling between the two levels and, on the right, the nonequilibrium approach based on two-photon processes, namely stimulated Raman scattering.** Only the latter scheme is used to realize the Dicke model.

In 2010, the superradiant transition of the open Dicke model was observed experimentally using neutral Rubidium atoms trapped in an optical cavity [11]. In these experiments, the coupling between the atoms and the cavity is not achieved by a direct dipole coupling between the two systems. Instead, the atoms are illuminated by an external pump, which drives a stimulated Raman transition. This two-photon process causes the two-level system to change its state from *down* to *up*, or *vice versa*, and emit or absorb a photon into the cavity (see **Fig 3**). Experiments showed that the number of photons in the cavity shows a steep increase when the pump intensity crosses a critical threshold. This threshold was associated with the critical coupling of the Dicke model.

In the experiments, two different sets of physical states were used as the *down* and *up* states. In some experiments [11–13], the two states correspond to atoms with different velocities, or momenta: the *down* state had zero momentum and belonged to a Bose-Einstein condensate, while the *up* state had a momentum equal to sum of the momentum of a cavity photon and the momentum of a pump photon [14]. In contrast, later experiments [15–16] used two different hyperfine levels of the Rubidium atoms in a magnetic field. The latter realization allowed the researchers to study a generalized Dicke model (see below). In both experiments, the system is time-dependent and the (generalized) Dicke Hamiltonian is realized in a frame that rotates at the pump's frequency.

## The generalized Dicke model and lasing

The Dicke model can be generalized by considering the effects of additional terms in the Hamiltonian of Eq 1 [5]. For example, a recent experiment [16] realized an open Dicke model with independently tunable rotating and counter-rotating terms. In addition to the superradiant transition, this *generalized* Dicke model can undergo a lasing instability, which was termed *inverted lasing* or *counter-lasing* [5]. This transition is induced by the counter-rotating terms of the Dicke model and is most prominent when these terms are larger than the rotating ones.

The nonequilibrium superradiant transition and the lasing instability have several similarities and differences. Both transitions are of a mean-field type and can be understood in terms of the dynamics of a single degree of freedom. The superradiant transition corresponds to a supercritical pitchfork bifurcation, while the lasing instability corresponds to a Hopf instability. The key difference between these two types of bifurcations is that the former gives rise to two stable solutions, while the latter leads to periodic solutions (limit cycles). Accordingly, in the superradiant phase the cavity field is static (in the frame of the pump field), while it oscillates periodically in the lasing phase [5].
